# Program adaptation by health departments

**DOI:** 10.3389/fpubh.2022.892258

**Published:** 2022-09-12

**Authors:** Louise Farah Saliba, Peg Allen, Stephanie L. Mazzucca, Emily Rodriguez Weno, Sarah Moreland-Russell, Margaret Padek, Ross C. Brownson

**Affiliations:** ^1^Prevention Research Center, Brown School at Washington University in St. Louis, St. Louis, MO, United States; ^2^Division of Public Health Sciences, Department of Surgery and Alvin J. Siteman Cancer Center, Washington University School of Medicine, Washington University in St. Louis, St. Louis, MO, United States

**Keywords:** adaptation, evidence-based intervention, implementation, public health practice, evidence-based decision making

## Abstract

**Introduction:**

The dissemination of evidence-based interventions (i.e., programs, practices, and policies) is a core function of US state health departments (SHDs). However, interventions are originally designed and tested with a specific population and context. Hence, adapting the intervention to meet the real-world circumstances and population's needs can increase the likelihood of achieving the expected health outcomes for the target population from the implemented intervention. This study identified how SHD employees decide to adapt public health programs and what influences decisions on how to adapt them.

**Materials and methods:**

SHD employees (*n* = 45) were interviewed using a qualitative semi-structured interview guide. Telephone interviews were audio-recorded and transcribed verbatim. The transcripts were consensus-coded and themes were identified using thematic analysis. Several themes aligned with the Model for Adaptation Design and Impact.

**Results:**

Data, outcomes, and health department evaluations influenced decisions to adapt a program (pre-adaptation), and reasons to adapt a program included organizational and sociopolitical contextual factors. SHD middle-level managers, program managers and staff, and local agencies were involved in the decisions to adapt the programs. Finally, the goals for adapting a program included enhancing effectiveness/outcomes, reach and satisfaction with the program; funding; and partner engagement. After SHD employees decided to adapt a program, data and evidence guided the changes. Program staff and evaluators were engaged in the adaptation process. Program managers consulted partners to gather ideas on how best to adapt a program based on partners' experiences implementing the program and obtaining community input. Lastly, program managers also received input on adapting content and context from coalition meetings and periodic technical assistance calls.

**Discussion:**

The findings related to decisions to adapt public health programs provide practitioners with considerations for adapting them. Findings reaffirm the importance of promoting public health competencies in program evaluation and adaptation, as well as systematically documenting and evaluating the adaptation processes. In addition, the themes could be studied in future research as mechanisms, mediators, and moderators to implementation outcomes.

## Introduction

In the U.S., state and local health departments deliver essential public health services, including preventing and controlling diseases with population-level approaches (1). The delivery of evidence-based interventions (EBIs; i.e., programs, practices, and policies) (2) is a core function of health departments (3). In some cases, an intervention found to be effective in one setting is less effective in a different setting or with a different population. In other cases, after an intervention is implemented, it may become ineffective or less effective than expected, yet is continued (4). In a study of state-level health department employees in 2018, 49% reported that programs sometimes, often or always continue when they should have ended (5).

Evidence-based interventions are typically designed and tested with a specific population within a specific context and research setting (6) and are generally implemented in settings different from the initial research testing context and population (7). Therefore, the implementation of an intervention or a program in the “real world” may benefit from adaptation. The definition of adaptation varies but is often defined as “modifying a program to meet the needs of the target population, local circumstances, or new contexts” (8). As illustrated by Stirman et al. (9), one example of population adaptation is an intervention originally developed for patients with a borderline personality disorder but being delivered to individuals with substance use disorder.

Program adaptation frameworks, summarized in a scoping review by Escoffery et al. (10), provide a comprehensive description of the stages and steps to guide the adaptation process. They provide a structure for identifying adaptable components of an intervention and its associated implementation strategies while maintaining fidelity to the core components. Other frameworks are available as tools for documenting the adaptation of programs (11) or implementation strategies (12). A conceptual model, the Model for Adaptation Design and Impact (MADI) (13), expands on earlier program adaptation frameworks and outlines the causal pathways of the adaptation elements that might impact the implementation outcomes. The model can be applied before implementing the adaptation, throughout implementation, and post-implementation.

Adaptation frameworks and models are tools designed for practitioners and researchers to aid planning, monitoring, evaluating, reporting, and studying the adaptation of interventions. These are comprehensive instruments and encompass different components in the adaptation process. Subsequently, the systematic use of these instruments strengthens the type 3 evidence generated in research and facilitates the implementation of interventions in the real world. Type 3 evidence provides information on the design and implementation of an intervention, the contextual circumstances in which the intervention was implemented, and how the intervention was received (14). In addition, understanding the contextual factors in which interventions are implemented and adapting interventions to different contexts in which they are implemented can increase the intervention fit (13) and the ability to scale up and transfer between contexts (15).

Yet, the process of adapting a program has inherent implementation challenges. There is no guarantee that an adapted program will generate the expected outcomes even when considering contextual factors and employing frameworks. Balancing adaptation with fidelity is a frequent struggle when implementing evidence-based public health interventions (16). Additionally, although the foundations for program adaptation have been established, the empirical knowledge about adaptation decisions is limited.

Several research teams have created models that can inform adaptation decision-making. Miller et al. (17) mentioned the presence of models representing steps for making adaptations to evidence-based practices (EBPs). However, these models do not provide an adequate description of how these phases interact. As a follow-up, Miller et al. (17) developed an adaptation decision-making framework that considers the inherent complexities throughout delineated decision points. The framework serves as guidance to conceptualize and document adaptations to EBPs in clinical contexts. Another framework for evidence-based decision-making (EBDM) in health systems was developed by Shafaghat et al. (18). The framework can be used to implement EBDM, especially in underdeveloped and developing countries. Although the framework is not specific to adapting EBIs, it could be of practical use in that process. Other authors addressed the decision-making process related to transferability in health promotion and disease prevention interventions (19). The PIET-T process model is meant as a decision-making and planning aid. It can be used to compare the context in which the intervention was developed and tested with the context to which the intervention will be transferred.

Our study sought to identify state health department (SHD) employees' decision-making processes around program adaptation to improve the effectiveness of public health programs. Using a qualitative description approach (20), we investigated how SHD section directors and program managers decide to adapt public health programs and what influences their decisions on whether and how to adapt programs. The findings provide public health practitioners with potential directions on the decision-making processes for adapting public health programs and inform *how* adaptations should be made, referred to as type 3 evidence (14, 21). In addition, this study illustrates a retrospective use of adaptation frameworks for future research, contributing to implementation science.

## Materials and methods

This study involved qualitative interviews with public health professionals working at state health departments. A qualitative description approach (20) was used and a codebook was developed to examine topics around adaptation of public health programs. Ethical approval for this study was granted by the Washington University in St. Louis Institutional Review Board (IRB# 201812062).

### Interview guide development

The interview guide questions aimed at understanding decision-making processes and factors related to mis-implementation of public health programs, i.e., ineffective programs that continue when they should have ended or effective programs that ended prematurely (22). The questions were developed based on the results of a previous national survey that examined programmatic decision-making in state health departments (5) and encompassed a socioecological structure (23). The interview guide questions asked about decision-making processes and the individual, organizational, and external factors related to programs that continued when they should have ended. The interview guide included broader, open-ended questions followed by specific questions to gain a detailed response from participants about adaptations, including the topics: who is involved in the decision to adapt a program, at what level are decisions made, how is it determined that a program needs to be adapted, what is the decision-making process when deciding that a program needs an adaptation, how is it determined what adaptations are appropriate, and what stakeholders are involved in adaptation decisions. Questions were pilot-tested and refined with the project's stakeholder advisory board, which included recently-retired state health department practitioners (see Supplementary materials).

### Participants and recruitment

Eight states were selected to recruit participants for this study. States were chosen to be representative of various population sizes and geographical locations, as well as high and low perceived levels of mis-implementation based on the results of a previous national survey (5). Each state's chronic disease program director was contacted *via* email to inform them that the research team would be inviting their staff to participate in interviews. During this contact, chronic disease program directors were also invited to participate and asked for recommendations for other interview participants. In one case, the program director requested that their staff not be contacted, resulting in the research team replacing this state with an alternative state.

Potential participants were then invited from each chosen state's chronic disease program. They were identified as having responded to the research team's previous national survey and/or based on recommendations from chronic disease directors or other staff. Participants were eligible if they served in a programmatic role in the chronic disease program (i.e., administrative staff were not eligible). Their recruitment details are found elsewhere (24, 25). In the end, the study team invited 152 individuals with valid email addresses by email, including 23 who explicitly refused to participate most often due to lack of time; others never responded to contact attempts or did not feel they had sufficient knowledge to answer the interview questions.

Interviews were conducted *via* phone between February and June 2019. The interview guide was provided to participants prior to the interview. The interviews were conducted by an all-female trained research team, including three graduate research assistants, the project manager, and a faculty member (including authors EW, SMR, and MP). Participants were offered a $40 gift card. Alternatively, for completing the interview, the interviewees were offered a $40 donation to a public health charity of their choice made from the project's budget on their behalf. All interviews were audio-recorded and professionally transcribed (Rev.com) for analysis in NVivo 12 (26). Field notes were taken during each interview to assist in the interpretation of interviews. Interview recruitment from each SHD ended when it was determined that few new points were heard or when we had already interviewed six employees from that particular SHD. Our team made this decision based on recommendations in the literature (27–29), our past experiences with qualitative research, as well as respect for the burden interviews placed on SHD chronic disease units.

### Data analysis

A qualitative description approach (20), which is suitable for studying the who, what, and where of events (30) and focuses on portraying data (31), was used in this study.

A codebook was developed a priori based on Stirman et al. (9) and stakeholder participation (32) frameworks to examine further program adaptation of public health programs undergoing mis-implementation. The first version of the adaptation codebook was created by two research team members (LF, ER) and presented to four research team members (PA, SM-R, MP, and SM) to receive feedback. Based on the team's feedback, a second version was created with fewer child codes. With version two of the codebook available, the first round of codebook pilot testing was conducted by the study team members (LF, ER, PA, SM-R, MP, and SM), where each study member coded a different transcript. The team then convened to discuss the issues they encountered while coding and discussed what needed to be addressed. Subsequently, the third version of the codebook was created, in which we added two child codes and details to the description of the child codes, and another round of pilot testing, following the same approach, was performed. The feedback after coding the transcripts using version three informed version four, where one of the previously-added child codes was removed and information added to the coding guidance for consistency among team members. The final codebook was the fourth iteration and had three parent codes for the type of adaptions, decision-making around adaptations, and stakeholder engagement. The first code, types of adaptation, had three child codes: contextual, content, and cultural modifications. The child codes and their descriptions were informed by Stirman et al. work (9). The second code was decision-making around adaptations, and it had two child codes: who is and who is not involved in adaptations and how is it determined to adapt. The third code was about stakeholder engagement and a spectrum for stakeholder participation (32). No child code was added to the third code and a definition of potential stakeholders in public health programs was included (33). Refer Supplementary materials to see the codebook.

For coding all transcripts, the six research team members were split into pairs, assigned a number of transcripts, and individually coded each transcript in NVivo using the final codebook. Each pair met to reach consensus on any discrepancies. If consensus could not be reached, a third coder reviewed the transcript and consulted with the two initial coders to come to an agreement. After completing transcript coding and consensus, two team members performed deductive thematic analysis, an appropriate method for a qualitative description approach (31). In the thematic analysis, the following steps were taken (34): (1) independently searched coded transcript texts for themes, (2) reviewed each other's draft themes, (3) reached consensus on a list of themes, (4) defined and named the themes, (5) produced theme reports with illustrative quotes for review by the full study team, and (6) noted which themes aligned with domains in MADI (13). This conceptual model can be used retrospectively, i.e., after adaptations are implemented, as a scaffolding for evaluating research questions, and as a guide to help identify potential mediators/moderators. The themes identified by the researchers aligned with two of the three MADI domains; from domain one, the “who” aligned with the data, and from domain two, “goal/reasons” and “systematic” aligned with the data. Researchers organized themes by topic into “deciding to adapt the program” and “adapting the program” to tell the story of program adaptation decision-making.

## Results

Table 1 presents the self-reported demographic characteristics of the participants. Forty-five SHD employees were interviewed from eight states with an average interview duration of 43 min (range 20–68 min). Most participants were program managers (64%), followed by section directors (22%), and females (all except one), who have been working in public health for an average of 15 years, and in their agency, for an average of 11 years.

**Table 1:** Demographic characteristics of state-level health department practitioners who participated in interviews on decision-making around program adaptation in the United States, 2019.

**Characteristics**	**Respondents (*N* = 45)** ***n* (%)^a^**
**Gender**	
Female	44 (98)
Male	1 (2)
**Position**	
Program Manager or Coordinator	29 (64)
Director overseeing multiple programs in a section, bureau, or division	10 (22)
Evaluator	2 (4)
Epidemiologist	2 (4)
Other (analyst, clinical care liaison)	2 (4)
**Time spent in current position (years)**	
≤ 5	26 (58)
6–10	9 (20)
≥11	7 (16)
**Time spent in current agency (years)**	
≤ 5	17 (38)
6–10	10 (22)
≥11	17 (38)
**Time spent in public health overall (years)**	
≤ 5	4 (9)
6–10	13 (29)
≥11	26 (58)

a Participants came from eight states representing all U.S. Census Bureau regions, including Northeast (three states), South (two states), Midwest (two states), and West (one state).

The themes related to the decision-making processes around adaptation for the topics *(1) deciding to adapt the program (pre-adaptation)* and *(2) adapting the program (during adaptation)* are presented below and found in Table 2.

**Table 2:** Topics and themes of the decision-making process for program adaptation of state-level public health programs in the United States, 2019.

**Topics**	**Themes**
Deciding to adapt the program	Decisions were directed by data, outcomes, and evaluation.
	Reasons included organizational and sociopolitical contextual factors.
	Decisions involved state health department middle-level managers, program managers and staff, and local agencies.
	Goals were to increase effectiveness/outcomes, reach, satisfaction with the program, funding, and partner engagement.
Adapting the program	Data and evidence were used to guide the changes.
	Program staff and program evaluator were engaged to guide how to adapt a program or its implementation.
	Partners and stakeholders were consulted to provide input on how to adapt a program.
	Systems and groups already in place were used to get input on how to adapt content and contexts.

### Topic: Deciding to adapt the program (pre-adaptation)

Participants reported different elements depicted by the themes when deciding to adapt a program. The themes included the factors influencing the decision to adapt a program, who was involved in this process, and the goals for those adaptations.

#### Decisions were directed by data, outcomes, and evaluation

Participants pointed out how much they relied on program evaluations or other data such as behavioral surveillance survey trends or return on investment calculations to guide their decision to adapt a program. Program evaluation data were the data on which participants relied for adaptation decision-making. Program evaluation data discussed by participants included quantitative participant surveys before and after implementation to detect changes in intervention clients' behavior or health status, as well as implementation process information from qualitative interviews.

“*So we do have an evaluator. We have an epidemiologist and then the program managers. And we all kind of serve as an umbrella hub to really ask those hard questions, ask the critical questions. We utilize our evaluation plans. We utilize our performance management plans. We really have those hard conversations with staff about, ‘The needle is not moving. Help us understand why,' or ‘Do we really need to go back and let go of this approach and try a new thing altogether?”[Participant 1]*

“*I think it really takes diligent and observant program directors or even the leaders of the DPPs [Diabetes Prevention Program], if they think about how... and they're evaluating their program itself, and who they're targeting and who they're reaching. I think it would take those types of people to go to the decision-maker and say, I think that this program isn't as effective as it could be, or that we could try to reach a different population in a different way. I also think that data speaks volumes, too, so if enough data can be shown that something needs to change, or what we're doing isn't working.” [Participant 2]*

“*And I think that's where evaluation comes in and we have a very strong evaluation team, and so we're able to look at from beginning to end, how does this work and is it effective and do we want to continue or do we need to adjust it to adapt it to the different needs in our communities*.” *[Participant 20]*

“*So I think any time that we are making those hard decisions about changes in the programing or letting something go or adding something new in, our conversations really now are, ‘At the end of a certain grant cycle, will we be able to show the outcomes that are needed to secure funds for [our state] or put us in the best place to receive those funds?” [Participant 1]*

#### Reasons included organizational and sociopolitical contextual factors

Other organizational and sociopolitical factors also affected the decision to keep a program and adapt it. Participants discussed the need to adapt programs to align with changing federal landscapes. The main example several participants described was the need to adapt recruitment processes and target recruitment populations for breast and cervical cancer screening or colorectal cancer screening after the Affordable Care Act expanded access to cancer screening and after many states expanded Medicaid eligibility.

“*Evaluation, outcomes on that was a priority, our return on investment was a priority, and then just overall community engagement, community support.” [Participant 3]*

“*When the new hypertension guidelines came out, we adopted them. I think it's based on funding and it's based on whether or not national funding partners decide, ‘We need to change that.” [Participant 4]*

“*So for example, prior to the Affordable Care Act, the women's cancer screening program, our primary objective was to help uninsured women. The amount of uninsured women decreased significantly post-ACA so we had to change the way we implement our program a little bit and that involved expanding our services to include under-insured women. So that was women who have insurance but who might have some difficulty paying for... like copays and deductibles associated with screening services. Particularly like follow-up diagnostic services.” [Participant 12]*

#### Decision involved SHD middle-level managers, program managers and staff, and local agencies

The decision to adapt a program was often made by SHD managers after discussion with team members, by program managers and their staff, or by SHD program teams in consultation with local agencies. Participants said most decision-making about adaptation happens at the program staff and program manager levels. Moreover, they mentioned that only programs with media attention or political considerations needed to go further up the SHD chain of command for decisions about modifying program content, target population, or recruitment or implementation strategies. Other participants said program staff and managers are involved in the adaptation decision-making in consultation with external partners, especially partners the SHD is contracting with to implement the program.

“*It would be myself; I pretty much oversee this program, and then also my program manager.” [Participant 5]*

“*Our chronic disease team meets. They're part of the same team, and so they have regular, ongoing staff meeting, communication meeting, all of that. They do their planning and they work together. And they're also connected with the larger provider community that administers such programs. And so I think that they're the ones that would be more in conversation and discussion about how programs are implemented and which ones are effective and what they hear from their colleagues and others.” [Participant 6]*

#### Goals were to increase effectiveness/outcomes, reach, satisfaction with the program, funding, and partner engagement

Participants often noted what they aimed to achieve when making a change. Some goals seemed to be directly related to addressing the lack of outcomes or due to sociopolitical and organizational reasons. Other goals seemed to be indirectly related and included the goals to enhance funding or partners' engagement and satisfaction by refreshing the program. Here are examples of the adaptation goal of increasing the reach of the program:

“*There was the building awareness and the outreach for recruitment of participants for the different counties. They were also very creative in identifying how one method of maybe recruitment or advertising of the program wasn't quite working, so they then tried a different way.” [Participant 7]*

“*And now, since we've refreshed the materials, they look new, they look different, they look exciting. So that has helped a little bit.” [Participant 8]*

### Topic: Adapting the program (during adaptation)

After deciding to adapt a program, four approaches were reported by the participants as part of the adaptation process. The following themes summarize the approaches used for selecting how to adapt a program and who is involved in this process.

#### Use of data and evidence to guide the changes

Participants mentioned using evaluation data and other evidence to inform their decisions when adapting their program. For this process, they relied on accessing available evidence from research, including evidence-based approaches and pilot projects, learning what was working in other states; and obtaining data from their program evaluations, including data from participant surveys, partner engagement surveys, and interviews with participants and partners.

“*An ineffective program I think is one that really isn't based in our evidence. I fully understand the importance of emerging evidence and research and piloting projects. …I rely a lot on evaluation and outcomes from evaluation initiatives that we do within our various programs. So, you know, just taking a look at the evidence, seeing what is working within our programming efforts, what may not be working, but adapting to that feedback too. So not just staying stagnant in our activities and initiatives, but learning from evaluation reports, learning from data collection and looking at data trends and making meaningful change that way.” [Participant 9]*

“*…So we're in the process of transitioning and that was all based on the evaluation results and there is... school-based programs are an evidence-based approach. ….We did a lot of research into this, the best ways to implement it. So we're in the process now of transitioning. It's a lot of work. We're just trying to implement it to the best of our knowledge, following all the evidence that's out there.” [Participant 10]*

“*The coalition member that was the lead on it, she found I believe it's a study done on library staff in Washington State. So she did some background research, learning what works, but again tweaking it so instead of library staff doing the screening and education it was hairstylists*.” *[Participant 21]*

#### Engagement with program staff and program evaluator

Program staff met regularly, e.g., weekly, monthly, or every 6 months, to discuss and plan the changes they wanted to make in the programs. Program managers and staff often brought in program evaluators, who were an important part of the process of deciding how to adapt a program. In some instances, leadership needed to approve the changes. In this process, they used data from evaluations to inform their changes.

“*The evaluator, our internal... our director of evaluation will usually do at least... as he's establishing the evaluation plans, he will meet frequently with the staff to go through that process of coming up with a good plan. He will meet often with stakeholders a number of times throughout the year and with program staff at least once a month as well. So, you know it's pretty frequent. And then, once an evaluation is underway then there's at least an annual review of what have we found? Where are we going? What do we... is there anything that we need to tweak? It's probably done more every six months actually.” [Participant 11]*

“*Well, honestly I think that's something in my unit that we're always thinking about. We have weekly meetings. Part of our meetings is how we can best approach programming and evidence base, and how we can meet our PM [performance management objectives]. So yeah, it's a continuous thought and conversation.” [Participant 22]*

“*Well, certainly during the planning process it's a lot. It's in a really short compressed timeframe. And then, once the funding is received and we start to really solidify the programs, then it's the program staff who will get together at least monthly, usually more frequently in the beginning but move to a monthly meeting to where they talk through what's happening. How are things going? What are we seeing? What aren't we seeing? Are there things that need to be tweaked?” [Participant 11]*.

#### Consultation with partners and stakeholders

In the process of making changes, program managers sometimes contacted their partners in other states. They paid attention to what was working in these states to rethink how they should do things in their SHD. Another approach used to decide on the changes was getting feedback from community stakeholders.

“*I do make, at the end of the day, decisions about what happens with the programs and the contracts that we make and the direction that we're moving in. But I would like to think that I sort of consult my peers as much as possible.” [Participant 12]*

“*The community came back and said no we want to do Zumba but the … program coordinator wanted to implant a yoga program and so the community said it is great that you are offering that it is better than nothing but if you really want to get this community up and moving Zumba is what you are going to need to offer, because people love to dance. So they [program coordinator] acted fast and started a Zumba program and that's what I saw there that I haven't seen in a lot of other programs.” [Participant 3]*

“*..we would often during our TA [technical assistance] calls at CDC, or in attending meetings or conferences across the state, across the country, we would find best practices of what other states were doing with similar funding, if not the same funding. We would also get feedback from the staff at the districts and also our partners. So we did partner engagement surveys to determine what was working, what wasn't working. We had calls often with our district-level folks, again to determine what was working, what wasn't working, and how we could kind of re-steer the ship to ensure that we were still meeting our goals and deliverables.” [Participant 13]*

“*I think here our leadership would generally rely on the programmatic folks and the division directors to sort of research and understand what other alternatives would be and to come up with a recommendation. I think if they felt like that wasn't working or wasn't the right way to go, they would reach out to other states to find out what they're doing.” [Participant 14]*

“*And obviously states are really willing to share, and that is really helpful because if somebody does find something that works in a new space, it really is helpful to kind of take their lessons learned, maybe avoid some of their pitfalls and their challenges that they had, but we always have to tweak it to the [state] landscape…we can't just pick up something that they've done and run with it because the partnerships are different, the infrastructures are different, the relationships are different.” [Participant 1]*.

#### Use of the systems and groups already in place to get input on how to adapt content and contexts

Participants mentioned SHD program team meetings, regular technical assistance calls, committees, and coalitions as opportunities to discuss, receive and provide feedback on the adaptation process. Some coalitions and committees were statewide, but participants also described instances where a local county or city coalition or committee provided input on which aspects of the program to adapt and how.

“*I have a statewide coalition and each one of my programs, our strategies have committee around it and we review the progress together and decide what's working out and what's not and how to either tweak it [program] or move it [the program into] something slightly different, I don't think it [program] was ever really discontinued unless there was no funding.” [Participant 15]*

“*We have monthly TA [technical assistance] calls. Our nurses do monthly TA calls with their assigned specially qualified health center, and the local health departments have a community health educator on our staff that is one of their TAs. A lot of times, they'll talk to their TA about an issue. If it's something that the TA can't address, then they'll bump it up to management. Then we'll discuss in grand rounds or sit in on a monthly call to see if we can address their concerns or figure out what needs to be done differently*.” *[Participant 16]*.

## Discussion

This study provides descriptions of state health department employees' decision-making processes around program adaptation of public health programs that others can apply when deciding whether or how to adapt a public health program. Program managers and section directors shared their decision-making approach to adapt a program. The decisions were (a) directed by data, outcomes, and evaluation, (b) influenced by reasons that included sociopolitical and organizational contextual factors, (c) involved SHD middle-level managers, program managers and staff, and the local agencies, and (d) aimed at increasing program effectiveness/outcomes, reach, satisfaction with the program, funding, and partner engagement. The program adaptation processes encompassed (a) using data and evidence to guide changes, (b) engaging with program staff and program evaluator, (c) consulting with partners and stakeholders, and (d) using systems and groups already in place to get input on how to adapt content and contexts. The findings provide practitioners and researchers with insights for decision-making around adapting public health programs.

For both topics, “deciding to adapt a program” and “adapting the program,” a link to evidence-based public health (EBPH) was identified as important. EBPH is defined as (35) “the process of integrating science-based interventions with community preferences to improve the health of populations.” Translating EBPH into practice can be achieved through implementing the following key components (36): making decisions on the basis of the best available, peer-reviewed evidence, using data and information systems systematically, applying program-planning frameworks, engaging the community in decision-making, conducting sound evaluation, and disseminating what is learned.

When deciding to adapt a program, interview participants mentioned EBPH approaches like using data, evaluating outcomes, and engaging the community in decision-making. Participants also involved programming staff in the decision to adapt the program, which is important since participatory decision-making when adapting a program may predict the impact of the changes (11). Additionally, the participation of program managers, staff, and local agencies in the adaptation decision-making process is aligned with the Framework for Reporting Adaptations and Modifications-Enhanced (FRAME) (11) and the MADI conceptual model (13). When deciding to adapt a program, participants' decisions were influenced by data and organizational and sociopolitical factors, which are examples of inner and outer contexts. These contexts are embedded in the implementation of EBPs (37) and the decision-making process of the evidence-based behavioral practice (38), affecting the implementation and sustainment of EBPs. Participants also noted their desired goals with the adaptations. The adaptation goals of increasing effectiveness/outcomes, reach, and satisfaction with the program are detailed in the FRAME framework (11) and could be related to achieving the EBIs' expected outcome. A study about fidelity and adaptation also found that desires to increase program reach and fit drove adaptation decisions (39). Additionally, other goals were identified that are not part of the FRAME framework (11) or the model for adaptation design MADI (13), including goals to enhance funding and partner engagement. These goals could be indirectly related to achieving the expected outcomes for the program or addressing sociopolitical and organizational factors.

When using data and the best available evidence to adapt a program, participants were applying EBPH skills (36), indicating a potential return of years of investment and efforts in building workforce capacity in EBPH (40). Another critical piece in the adaptation process is the involvement of stakeholders to inform the adaptation and implement it. Stakeholders have a well-established presence in the adaptation process (11, 13, 41). As discussed by participants, state and local partners and other stakeholders have contextual knowledge important in making decisions on whether or how to adapt a program. Furthermore, participants collaborated with partners to learn about programs that work or consulted with them to adapt programs, illustrating and reinforcing the importance of communication and inputs in the dissemination and implementation cycles (41). Interestingly, participants said they benefited from structures in place to get input from their partners, like periodic technical assistance calls or coalition meetings.

Our findings are aligned with the interview guide framework (22) used for this study, capturing different levels of the socioecological model (23) in the processes of adapting programs and generating type 3 evidence (14). For example, the inputs received from partners and stakeholders, the involvement of SHD middle-level managers, program managers, staff, and evaluators are linked to the interpersonal level; the organizational reasons are connected to the organizational level; and the sociopolitical contextual factors are related to the sociopolitical level.

The findings reinforce the importance of promoting public health capacities in program evaluation and adaptation. Participants often mentioned that adaptation needs were identified once someone with evaluation skills was involved in the program, highlighting program evaluation's critical contribution to deciding whether to adapt and successfully adapt a program (8). Training is an important component of public health workforce development (36). Maintaining program evaluation capacity through hiring practices and on-the-job training continues to be important (42). A recent national survey identified training needs in change management (i.e., modifying programmatic practices in consideration of internal and external changes) and stakeholder development of a vision for a healthy community (43) as well. Increasing access to adaptation training sessions, technical assistance, and tools could advance staff program adaptation expertise (44). Additionally, health departments should evaluate the impact of training programs on skill attainment and use (45).

Deliberate efforts are needed to promote program adaptations as a systematic and evidence-informed practice. Having established procedures for documenting translation and adaptation can help recognize whether the adaptation is effective (46). To implement those practices, researchers and practitioners can take advantage of existing resources, including frameworks for guiding the adaptation process (10, 47) and reporting adaptations (11) and a conceptual model for linking adaptation elements to outcomes (13). Furthermore, step-by-step methods (48) for identifying the intervention's essential elements to preserve its efficacy and effectiveness could also be employed. Other resources can be utilized, like the Dissemination and Implementation Models online tool (49), which displays models and tools, and other resources being created that could be put into practice, like the adaptome data platform (50). Enabling a platform that can be easily accessed when adapting a program could accelerate the progress and success of program adaptation.

The study has a few limitations. Our data are from eight states; although we aimed for representativeness in the selection strategy of the participating states, it is still a limitation. Differences in how programs are governed in different states and territories and other organizational and environmental characteristics might have led to variations in the approaches used to adapt programs. Another limitation was that program effectiveness was based on the participants' perceptions and it was not investigated if the changes resulted in the expected outcomes for the programs. Adaptation decision-making was only a portion of each interview, so our findings are not a comprehensive view of adaptation but inform this area of research. Additionally, we were not able to analyze adaptation decision-making power by equity-relevant subgroups (e.g., by race/ethnicity) since we did not know which partner groups were from marginalized communities.

The findings have implications for public health practice, policy and research. First, the results depict the decision-making process for adapting programs in state health departments, providing practitioners with potential directions for adapting public health programs. Second, the findings reinforce the importance of promoting public health capacities in program evaluation and adaptation, indicating that agencies' policies could support more investments and plans to enhance skills in these areas. Third, documenting the adaptation process is important (11) and should be pursued by practitioners and supported by funders when appropriate. Fourth, research should be conducted to determine if the adapted programs are effective compared to the original ones (8) and if the adapted programs meet desired goals (e.g., increasing reach). Fifth, it is important to ensure that voices from racial and ethnic identities and marginalized communities are included in adaptation decision-making. Lastly, this study identifies potential moderators, mediators, and mechanisms to promote adaptability (51) that might impact implementation outcomes (13, 52). The processes' elements could be represented in a causal pathway (53) and tested empirically in a socioecological multilevel approach (23) throughout the different program implementation phases (54).

Using a qualitative analytical method (20), we investigated how state health department unit directors and program managers decide to adapt public health programs to enhance reach and impact and what influences how they do so. State health department staff employed a variety of approaches when making decisions about adapting programs. The methods included using data, evaluation, and evidence; considering the department's internal and external reasons; envisioning goals for the modifications; involving SHD employees, partners, and stakeholders; and using systems and groups to gather input on whether and how to adapt content and contexts. Our results contribute insights into the decision-making process on how to adapt programs generating type 3 evidence (i.e., “how”) and illustrating a retrospective use of adaptation frameworks in research. Our findings support continued development of public health workforce capacities and the systematic documentation and evaluation of the adaptation process. Lastly, the study points out elements to be further explored as mechanisms, mediators, and moderators of implementation outcomes. Our results inform future research to support practice and policy development and assist public health programs in achieving the expected population-level outcomes.

## Data availability statement

The datasets presented in this article are not readily available because of participants confidentiality concerns regarding the raw data, but data summaries are available upon reasonable request. Requests to access the datasets should be directed to LF: l.farah@wustl.edu.

## Ethics statement

The studies involving human participants were reviewed and approved by Washington University in St. Louis Institutional Review Board. Written informed consent for participation was not required for this study in accordance with the national legislation and the institutional requirements.

## Author contributions

RB conceptualized, designed the study, and outlined the introduction section. SM, SM-R, ER, PA, MP, and RB developed the interview guide. SM-R, ER, and MP conducted the interviews. SM, SM-R, LF, ER, PA, and MP coded the interviews' transcripts. ER and LF synthesized the themes and reviewed the first draft. ER drafted the methods section. LF drafted the other sections. PA and SM contributed to the discussion of the findings. All authors read, contributed to manuscript revision, and approved the submitted version.

## Funding

This project is funded by the National Cancer Institute of the National Institutes of Health (R01CA214530). Additional support came from National Cancer Institute (P50CA24431, T32CA190194), the National Institute of Diabetes and Digestive and Kidney Diseases (P30DK092950, P30DK056341, and R01DK109913), the Centers for Disease Control and Prevention (U48DP006395), and the Foundation for Barnes-Jewish Hospital. The findings and conclusions in this manuscript are those of the authors and do not necessarily represent the official positions of the National Institutes of Health or the Centers for Disease Control and Prevention.

## Conflict of interest

The authors declare that the research was conducted in the absence of any commercial or financial relationships that could be construed as a potential conflict of interest. The handling editor MM declared a past collaboration with the author RB.

## Publisher's note

All claims expressed in this article are solely those of the authors and do not necessarily represent those of their affiliated organizations, or those of the publisher, the editors and the reviewers. Any product that may be evaluated in this article, or claim that may be made by its manufacturer, is not guaranteed or endorsed by the publisher.
