# Supplementary material for: Program adaptation by health departments
Source: Front Public Health. 2022 Sep 12;10:892258. doi: 10.3389/fpubh.2022.892258 (PMC9512313; doi:10.3389/fpubh.2022.892258)
Supplement: Supplementary file 2 [file Data_Sheet_2.docx]

| **Node** | **Code Name** | **Description** |
| --- | --- | --- |
| 1.0 | Type of adaptation | |
| 1.1 | Contextual Modifications | **Contextual** modifications include format, setting, channel of delivery and intervention recipients, and are about ‘setting the stage’ for an intervention to be delivered. |
| 1.2 | Content Modifications | **Content** modifications include substantive changes to the intervention (e.g., omission or addition of an educational module, changes to which aspects of the topic are addressed), minor tailoring/tweaking/refining of the intervention that leaves major intervention principles intact (e.g., modifying language, creating slightly different handouts), changes in which actions the intervention recipients are expected to take, and/or shortening the intervention duration. |
| 1.3 | Cultural Modifications | **Cultural modifications** are the systematic modification of an evidence-based intervention (EBI) to consider language, culture, and context in such a way that it is compatible with the client’s cultural patterns meanings and values. (e.g., incorporating culturally relevant examples of healthy behaviors, modifying language for cultural fit, including family in the intervention, incorporating relevant spiritual elements into content and delivery, changes images or videos used in the intervention to include representatives from the cultural/ethnic target audience, relying more on illustrations than text to convey information in cultural groups with lower literacy). |
| 2.0 | Decision-making around adaptations | |
| 2.1 | Who and who is not involved | Who and who is not involved in the decision to adapt a program (i.e., at what level are decisions made). This could include stakeholders and/or internal SHD staff. |
| 2.2 | How its determined to adapt | How it is determined that a program needs to be adapted. What is the decision-making process when deciding that a program needs an adaptation, how is this conclusion reached (ex., review of evaluation results, funder requires, etc.). |
| 3.0 | Stakeholder engagement | |
| 3.0 | Stakeholder engagement | Stakeholders are individuals and organizations that have an interest in or are affected by a public health program and/or its results. Engagement with stakeholders can happen at different stages in the adaptation process and levels including informing, consulting, involving, collaborating, and empowering stakeholders. |

**Coding Guidance**

- Text can be coded to more than one node.
- Cultural modifications should be double coded as either content or contextual codes also depending on the type of adaptation.
- When a stakeholder is involved in decision-making around adaptations it should be double coded in nodes 2.1 and 3.0.
- Prioritize coding for the child node. Code only to the parent node if the quote doesn't fit in the child node.
- Code the entire response after an interviewer’s question including punctuation.
- Code the interviewer’s question when the participant’s response is missing necessary content for understanding.
- Code any changes to programs described even if they are not explicitly called “adaptations.”
- Do not code descriptions of hypothetical adaptations.
